# People With Autism Spectrum Conditions Make More Consistent Decisions

**DOI:** 10.1177/0956797617694867

**Published:** 2017-06-21

**Authors:** George D. Farmer, Simon Baron-Cohen, William J. Skylark

**Affiliations:** Department of Psychology, University of Cambridge

**Keywords:** autism, decision making, attraction effect, rational choice, open data, open materials, preregistered

## Abstract

People with autism spectrum conditions (ASC) show reduced sensitivity to contextual stimuli in many perceptual and cognitive tasks. We investigated whether this also applies to decision making by examining adult participants’ choices between pairs of consumer products that were presented with a third, less desirable “decoy” option. Participants’ preferences between the items in a given pair frequently switched when the third item in the set was changed, but this tendency was reduced among individuals with ASC, which indicated that their choices were more consistent and conventionally rational than those of control participants. A comparison of people who were drawn from the general population and who varied in their levels of autistic traits revealed a weaker version of the same effect. The reduced context sensitivity was not due to differences in noisy responding, and although the ASC group took longer to make their decisions, this did not account for the enhanced consistency of their choices. The results extend the characterization of autistic cognition as relatively context insensitive to a new domain, and have practical implications for socioeconomic behavior.

People with autism spectrum conditions (ASC) often show atypical performance on tasks that require processing of local information independently of its context (Behrmann, Thomas, & Humphreys, 2006; Frith, 1989; Happé & Frith, 2006). For example, people with ASC are better than control participants at finding figures embedded in complex shapes, their visual search is less affected by the number and similarity of distractors, and they often fail to take semantic context into account when pronouncing homographs (see Happé & Frith, 2006, for a review). Nonclinical samples scoring high on measures of autistic traits display a similar pattern of performance (e.g., M. E. Stewart, Watson, Allcock, & Yaqoob, 2009). This reduced impact of context may reflect an inability to integrate information into a coherent whole (Frith, 1989) but may also be understood solely in terms of a superior ability to process local information (Plaisted, Saksida, Alcántara, & Weisblatt, 2003).

We investigated whether the reduced context sensitivity that characterizes ASC extends to decision making. Decision making is a fundamental cognitive operation that has received relatively little attention from autism researchers (Davis & Plaisted-Grant, 2015; Luke, Clare, Ring, Redley, & Watson, 2012). Most previous studies have focused on how people with ASC represent and evaluate probabilities and rewards, often using tasks in which the decision maker must learn the payoffs and probabilities of different options by making a series of choices and receiving feedback (e.g., Mussey, Travers, Klinger, & Klinger, 2015). We took a different approach by examining whether autistic traits correlate with altered context sensitivity in a riskless choice task, in which the participant simply selects the best alternative on the basis of explicitly stated attribute values.

Conventional accounts of rational choice dictate that a person’s preference between two items be independent of the other options on offer: If one prefers salmon to steak, this should not change just because frogs’ legs are added to the menu (Luce & Raiffa, 1957). However, the choices of neurotypical adults are heavily influenced by the composition of the choice set; rather than being based on an independent assessment, the attractiveness of a given option depends on how it compares with the other values that are simultaneously present (Huber, Payne, & Puto, 1982; Simonson, 1989; Tversky, 1972). One of the most striking examples of this phenomenon is the *attraction effect*, which arises when people choose between two options, A and B, that “trade off” two dimensions—for example, two USB drives that respectively have lower capacity but higher longevity and higher capacity but lower longevity. When the choice set includes a third, “decoy” option that is fractionally worse than A on both dimensions, people very rarely choose the decoy, but its presence boosts the tendency to choose A rather than B—and vice versa if the decoy targets option B (Fig. 1, top panels). This kind of context-induced preference reversal occurs in many domains (e.g., Farmer, El-Deredy, Howes, & Warren, 2015), has been extensively modeled (e.g., Trueblood, Brown, & Heathcote, 2014), and is used by marketers to influence consumer behavior (Ariely, 2009).

**Fig. 1.**
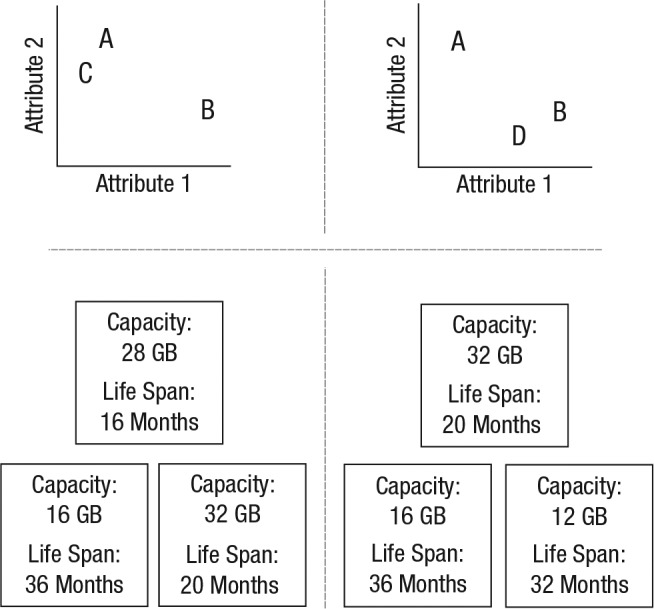
An illustration of the configuration of choice sets that elicit the attraction effect (top) and example trials from the experiment (bottom). In the top panels, A and B are options that trade off two positive attributes; C and D are decoys. Given a choice among A, B, and C, people typically choose A, but when offered a choice among A, B, and D, they prefer B. The bottom panels illustrate typical trials from the experiment—in this case, choices among USB drives. In the bottom left panel, the option on the lower right is the target, the option on the lower left is the competitor, and the option on the top is the decoy. In the bottom right panel, the option on the lower left is the target, the option on the lower right is the decoy, and the option on the top is the competitor.

If the tendency of people with ASC to prioritize local information and to be relatively insensitive to the other elements of a stimulus array extends to decision making, then they should be less influenced by decoy options and make fewer context-induced preference reversals. Correspondingly, we hypothesized that adults with ASC would make more consistent choices—indicative of a more rational, independent valuation of alternatives—than would neurotypical adults. This possibility is important because choice consistency is regarded as normative in conventional economic theory, so reduced context sensitivity would provide a new demonstration that autism is not in all respects a “disability” (Baron-Cohen, 2000). More important, context effects on choice speak to the nature and basis of autistic cognition. Many studies of altered context sensitivity among people with ASC focus on perceptual tasks, such as pitch discrimination, visual search, and motion-coherence detection, with corresponding theoretical frameworks that emphasize “low-level” processes, such as enhanced perceptual discrimination or altered magnocellular sensitivity (see Happé & Frith, 2006, for a review). Altered preferences in a choice task involving verbally described consumer products would suggest the need for a broader characterization and integrated theorizing across levels and domains of processing (Davis & Plaisted-Grant, 2015; Pellicano & Burr, 2012). Finally, the possibility of reduced contextual influence has practical implications for the economic and social functioning of people with ASC: Attraction-effect decoys influence many real-world decisions (e.g., Doyle, O’Connor, Reynolds, & Bottomley, 1999), and reduced decoy sensitivity among people with ASC would affect their financial, consumer, political, and relationship choices.

## Method

We conducted two studies. Our main experiment, which we refer to as the ASC study, compared adults diagnosed with ASC and a control group. In an additional experiment, which we refer to as the AQ study, we compared participants from the general population who scored in the bottom (*n* = 176) and top (*n* = 194) deciles of the Autism-Spectrum Quotient (AQ; Hoekstra et al., 2011), one of several self-report questionnaires that support the existence of a spectrum of autistic traits in the general population.

All participants completed a decision-making task adapted from previous studies of the attraction effect (Noguchi & Stewart, 2014). They also completed the International Cognitive Ability Resource (ICAR; Condon & Revelle, 2014) and the short-form Autism-Spectrum Quotient (AQ-Short), which we refer to as the AQ. The research was approved by the University of Cambridge Psychology Research Ethics Committee, and all participants provided informed consent.

### ASC study: comparing ASC participants with control participants

#### Participants

In this preregistered study, 90 ASC participants were recruited via the University of Cambridge Autism Research Centre, which provides a widely used participant pool for both lab and online studies (e.g., Uzefovsky, Allison, Smith, & Baron-Cohen, 2016). To register as an ASC participant in this database, participants must self-report a diagnosis of an ASC, specify its type (e.g., autism, Asperger’s syndrome), and report the kind of clinician who diagnosed them (e.g., psychiatrist) and the clinic or hospital where the diagnosis took place. (In lab-based studies that we have conducted using this participant pool, the vast majority of participants have been able to bring written evidence of diagnosis.) In the present study, we excluded 8 participants who, despite being invited to take part via the database mailing list, indicated that they did not have an official diagnosis of an ASC. (The presence of any residual nongenuine participants in the ASC group would simply mean that our tests of group differences are conservative.) Our final sample comprised 37 males, 52 females, and 1 participant who preferred not to report his or her gender; 70 participants were residents of the United Kingdom, 18 were residents of the United States, and 2 preferred not to say. Their ages ranged from 18 to 71 (*M* = 43.11, *SD* = 13.73), their ICAR scores ranged from 1 to 16 (*M* = 10.83, *SD* = 3.98), and their AQ scores ranged from 62 to 112 (*M* = 92.5, *SD* = 10.57). An additional 3 people were excluded for failing more than two catch trials (which served as an attention and comprehension check, as described in the Design and Procedure section; this exclusion criterion was set in advance).

Two hundred twelve control participants were recruited via the PureProfile market-research platform (www.pureprofile.com). This group comprised 89 males and 123 females; 169 participants were residents of the United Kingdom, and 43 were residents of the United States. Their ages ranged from 19 to 71 (*M* = 43.88, *SD* = 13.5), their ICAR scores ranged from 1 to 16 (*M* = 7.25, *SD* = 3.66), and their AQ scores ranged from 41 to 91 (*M* = 65.20, *SD* = 10.04). An additional 41 people were excluded for failing more than two catch trials. Control participants were group-matched with the ASC sample for age, gender, and country of residence.

Full demographic information for the ASC and control groups, including correlations between variables, is in the Supplemental Material available online (Tables S3–S5). The sample size was based on our decision to recruit as many ASC participants as possible and then calculate the number of control participants necessary to provide approximately 70% power to detect a difference between groups given the difference in proportions of consistent choices observed between the low- and high-AQ groups in the AQ study, which was conducted first (higher power would require unfeasibly large numbers of control participants; Faul, Erdfelder, Buchner, & Lang, 2009). Testing took place online using custom-written software; only participants who were at least 18 years old, whose IP addresses had not already been registered, and who provided complete data were included in the final sample.

#### Design and procedure

The choice task was adapted from previous studies of the attraction effect (Noguchi & Stewart, 2014). Participants saw 10 pairs of products; the products in each pair differed on two dimensions (Fig. 1). Each pair was presented twice, once with a decoy that targeted one product and once with a decoy that targeted the other; in addition, on six catch trials, one of the three products was clearly superior to the two others. One trio of products (target, competitor, and decoy) was presented on each trial. (See Table S1 in the Supplemental Materials for a list of all the stimuli.) Each trial was presented on a separate Web page headed by text describing the products’ attribute values, below which the three product descriptions were arranged in a triangle. Allocation of items to locations was random. Participants clicked the item they thought was best and advanced to the next choice. Trial order was random except that a product category was not displayed for a second time until all 10 product pairs had been displayed once. Cognitive ability was measured using the Matrix Reasoning, Three-Dimensional Rotation, Verbal Reasoning, and Letter and Number Series components of the ICAR, a validated measure of general cognitive ability that is tailored for online testing and comprises 16 items, each scored 0 for incorrect and 1 for correct (Condon & Revelle, 2014). Autistic traits were measured using the 28 items of the AQ-Short, each scored from 1 to 4 (Hoekstra et al., 2011). The order of the tasks (choice task, ICAR, and AQ) was randomized.

### AQ study: comparing low-AQ and high-AQ groups

We also compared people who had low and high levels of autistic traits and were drawn from the general population. Obtaining a pattern among individuals with high AQ scores similar to that observed in the clinical sample would provide converging evidence for a link between autistic traits and altered decision making, and would generalize the importance of this finding to a larger section of the population.

#### Participants

There were two versions of the AQ study. Version 1 was conducted first; Version 2, which used different stimuli and participants, was run as a replication study after the data from Version 1 were analyzed. Because the results are not modulated by version, we present the results of the combined analysis to give the best overall estimate of the effects; results for the separate versions are shown in Figures S8 and S9 of the Supplemental Material. In Version 1, 965 participants completed the AQ-Short; 81 from the bottom decile of AQ scores and 94 from the top decile of AQ scores returned to complete the main task. In Version 2, 1,008 participants completed the AQ-Short; 95 from the bottom decile and 100 from the top decile returned to complete the main task. Across both versions, a total of 18 additional participants completed the choice task but were excluded for failing more than two catch trials. Overall, the low-AQ group comprised 85 males, 90 females, and 1 participant who preferred not to report his or her gender; their ages ranged from 19 to 75 years (*M* = 35.73, *SD* = 11.86), their ICAR scores ranged from 1 to 16 (*M* = 8.18, *SD* = 3.48), and their AQ scores ranged from 37 to 54 (*M* = 49.48, *SD* = 3.77). The high-AQ group comprised 112 males and 82 females; their ages ranged from 20 to 69 (*M* = 35.55, *SD* = 10.48), their ICAR scores range from 1 to 16 (*M* = 9.53, *SD* = 3.32), and their AQ scores ranged from 79 to 107 (*M* = 85.24, *SD* = 5.38). (Full demographic information, including correlations between variables, is available in Tables S6–S9 in the Supplemental Material.) The sample sizes were chosen to achieve more than 80% power to detect a medium-sized effect (*d* = 0.5) in simple between-groups comparisons (Faul et al., 2009). Participants were recruited via Amazon’s Mechanical Turk (Buhrmester, Kwang, & Gosling, 2011).

#### Design and procedure

Participants completed the AQ-Short; those in the top and bottom deciles were invited back to complete the choice task and ICAR (task order was randomized). Version 1 of the study used the same stimuli as the ASC study; Version 2 used different products, adapted from Noguchi and Stewart (2014). (Tables S1 and S2 in the Supplemental Material list the stimuli used.)

## Results

### ASC study: context effects among people with ASC

Responses to each product pair were placed into one of four categories. *Consistent choices* were those in which the decision maker chose the same item on both presentations of a particular product pair. *Attraction-effect preference reversals* were cases in which the person’s selection switched when the decoy changed (the person chose A when the decoy targeted A and chose B when it targeted B). *Non-attraction preference reversals* were preference reversals in which the person chose the nontarget options on both presentations (e.g., the person chose A when B was the target and chose B when A was the target). *Decoy selections* were cases in which the person chose the decoy on one or both presentations of a given product pair.

**Fig. 2.**
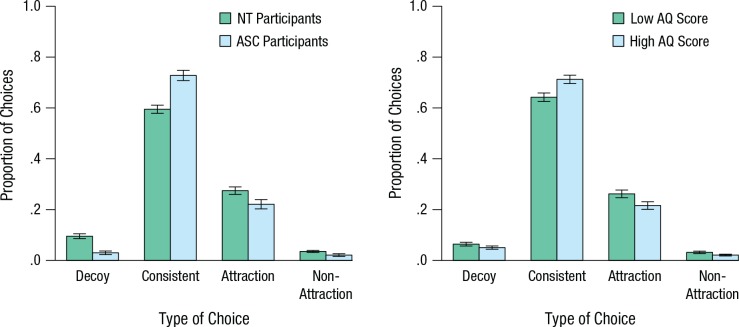
Mean proportion of choices from among four possible types of choices. The left panel shows results for the main study, which compared participants with autism spectrum conditions (ASC) with neurotypical (NT) adults. The right panel shows results for the additional study, which compared participants who scored low and high on the Autism-Spectrum Quotient (AQ). Error bars show ±1 *SE*.

As the left panel of Figure 2 shows, the ASC group made more consistent choices than did the control group, *t*(203.9) = 5.15, *p* < .001, *d* = 0.60, 95% confidence interval (CI) = [0.34, 0.85], and showed fewer preference reversals—attraction-effect reversals: *t*(206.4) = 2.27, *p* = .024, *d* = 0.26, 95% CI = [0.01, 0.51]; non-attraction reversals: *t*(194.6) = 2.12, *p* = .035, *d* = 0.25, 95% CI = [0.00, 0.50]. They also made fewer decoy selections, *t*(294.9) = 5.46, *p* < .001, *d* = 0.53, 95% CI = [0.28, 0.78]. We had a surprisingly high proportion of females in our ASC sample, but the choices of male and female ASC participants differed very little (see Table S10 in the Supplemental Material), which indicates that the sample’s gender composition does not affect the representativeness of the results for the ASC population.

Our primary analysis tested these effects more rigorously with a series of mixed-effects logistic regressions that examined the effects of group on choice, controlling for age, gender, and cognitive ability (regressions were conducted using lme4 for R; Bates, Mächler, Bolker, & Walker, 2015). The fixed-effects predictors were participant group (control = 0, ASC = 1), ICAR score, gender (female = 0, male = 1), and age. In all analyses, the predictors were standardized prior to each regression, and we included random intercepts for participant and product pair and by-product random slopes for the effects of group, age, gender, and ICAR score, thereby allowing the effects of these variables to differ across product pairs; random effects were uncorrelated (Barr, Levy, Scheepers, & Tily, 2013; dropping the random slopes did not impair model adequacy and led to virtually identical results). All confidence intervals (CIs) are 95% Wald CIs; the regression coefficients are presented graphically in Figure S1 of the Supplemental Material.

Our first regression contrasted consistent choices (coded 1) with preference reversals (coded 0). Participants in the ASC group made more consistent choices than did control participants, *b* = 0.200, 95% CI = [0.059, 0.341], *p* = .005, demonstrating reduced context sensitivity and a more rational decision-making style. There was little effect of age, *b* = 0.093, 95% CI = [−0.046, 0.231], *p* = .190; gender, *b* = 0.078, 95% CI = [−0.062, 0.217], *p* = .274; or cognitive ability, *b* = 0.096, 95% CI = [−0.062, 0.253], *p* = .233.

We also contrasted decoy selections (coded 1) against all other choice types (coded 0). Decoy selections are rare and represent a form of noisy responding/inattention (the decoy is manifestly worse than the target option). This kind of error was negatively related to general cognitive functioning, *b* = −0.679, 95% CI = [−0.944, −0.414], *p* < .001, and was less prevalent among ASC participants than control participants, *b* = −0.371, 95% CI = [−0.640, −0.102], *p* = .007, a result consistent with the higher attention-check failure rate in the control group. Decoy selections did not depend on age, *b* = 0.007, 95% CI = [−0.234, 0.248], *p* = .954, or gender, *b* = 0.036, 95% CI = [−0.214, 0.283], *p* = .784.

Finally, we contrasted attraction-effect and non-attraction preference reversals. Non-attraction-effect choices were very rare and, like decoy choices, they likely reflect noisy responding; they were more common among people with lower cognitive ability, *b* = 0.689, 95% CI = [0.357, 1.021], *p* < .001, but did not differ between the ASC and control groups, *b* = −0.037, 95% CI = [−0.381, 0.307], *p* = .834, and were unrelated to age, *b* = 0.014, 95% CI = [−0.295, 0.324], *p* = .929, or gender, *b* = −0.229, 95% CI = [−0.528, 0.069], *p* = .132.

### AQ study: context effects among high- and low-AQ groups in the general population

The choice proportions for low-AQ and high-AQ participants are plotted in the right panel of Figure 2 and show an attenuated version of the pattern found in the ASC study: High-AQ participants made more consistent choices than did low-AQ participants, *t*(366.6) = 3.00, *p* = .003, *d* = 0.31, 95% CI = [0.11, 0.52], and showed fewer attraction-effect preference reversals, *t*(366.9) = 2.16, *p* = .031, *d* = 0.22, 95% CI = [0.02, 0.43], although both groups exhibited a clear context effect. The high-AQ group also made slightly fewer decoy selections, *t*(355.3) = 1.40, *p* = .163, *d* = 0.15, 95% CI = [−0.06, 0.35], and non-attraction preference reversals, *t*(328.8) = 1.80, *p* = .072, *d* = 0.19, 95% CI = [−0.02, 0.40].

When applying the regression analyses to this study, we included study version and its interactions with all other fixed-effects variables to examine the consistency of the findings across participant samples and stimulus sets. In the same way as for other variables, version was standardized prior to each regression, and the interaction terms were computed by multiplying the standardized predictors. (No random effects involving version or its interactions were computed because all participants and all product pairs arose only for one version.) None of the effects were modulated by version, and excluding version and its interactions made no difference to the results. (The full set of regression coefficients for these terms is shown in Fig. S7 in the Supplemental Material.)

High-AQ participants were more likely to make consistent choices than were low-AQ individuals, *b* = 0.159, 95% CI = [0.011, 0.307], *p* = .035. In addition, consistent choice was positively related to age, *b* = 0.171, 95% CI = [0.037, 0.305], *p* = .013, and more common among males than females, *b* = 0.243, 95% CI = [0.110, 0.377], *p* < .001; it was also weakly related to cognitive ability, *b* = 0.148, 95% CI = [−0.006, 0.302], *p* = .060. The tendency to choose the decoy did not differ between the low-AQ and high-AQ groups, *b* = −0.033, 95% CI = [–0.241, 0.176], *p* = .758, but, as in the ASC study, it was negatively related to general cognitive functioning, *b* = −0.424, 95% CI = [−0.623,−0.224], *p* < .001; it was also slightly lower in males than in females, *b* = −0.201, 95% CI = [−0.398, −0.005], *p* = .044, but was independent of age, *b* = −0.075, 95% CI = [−0.287, 0.138], *p* = .490. Finally, non-attraction-effect choices were again very rare and more prevalent among people with lower cognitive ability, *b* = 0.538, 95% CI = [0.205, 0.870], *p* = .002. The proportion of non-attraction reversals was not affected by AQ, *b* = 0.013, 95% CI = [−0.271, 0.297], *p* = .928, or by gender, *b* = −0.220, 95% CI = [−0.508, 0.068], *p* = .134, or by age, *b* = −0.121, 95% CI = [−0.429, 0.186], *p* = .439.

The demographics questions asked participants whether they had ever been diagnosed with an ASC (response options: “yes,” “no,” “prefer not to say”). Seven of the 194 (3.6%) participants in the high-AQ group answered “yes,” whereas none of the 176 in the low-AQ group did so. Excluding these participants made little difference to the estimated coefficients, but the confidence intervals increased, including the confidence intervals for the effect of participant group on the tendency to make consistent choices versus preference reversals, *b* = 0.155, 95% CI = [0.006, 0.304], *p* = .041. A further 13 high-AQ and 2 low-AQ participants selected “prefer not to say”; excluding these participants led to a weaker effect, *b* = 0.123, 95% CI = [–0.026, 0.272], *p* = .107.

Thus, independently of the effects of age, gender, and general cognitive performance, people scoring high on the AQ made more consistent choices than did those with low AQ scores. The effect was weaker than in our ASC study, may reflect the presence of people with ASC in the high-AQ sample, and could have been driven by different mechanisms (Gregory & Plaisted-Grant, 2016), but it provides converging evidence for an association between autistic traits and a reduction in context sensitivity during choice.

### Additional analyses

We probed three possible contributors to the enhanced rationality of the ASC group’s choices and the high-AQ group’s choices: a drive for greater internal consistency, a reduction in noisy responding, and a slower, more deliberative decision-making style. Full results for these additional analyses, including regression coefficients for demographic control variables, are provided in the Supplemental Material (Figs. S2–S6 and S10–S14).

### First choices

One possible reason for a reduction in context-induced preference reversals is that people remember their own past choices and strive to be consistent. If the ASC group had better memory or a stronger drive for consistency than the control group, then a change in the attributes of the decoy between successive presentations of a product pair would have less effect on their choices, as we found, but this would apply only to within-subject preference reversals: The memory and consistency mechanisms would reduce the ASC participants’ tendency to switch their preference when the context changed, but they would be just as susceptible as control participants to the effects of the decoy when they first encountered a given pair of products. However, analyzing responses to the first occurrence of each product category revealed the same pattern as our main analysis: Decoy selection was less common among ASC participants than among control participants, *b* = −0.410, 95% CI = [−0.742, −0.078], *p* = .016, and, more important, people with ASC were less likely than control participants to choose the target item rather than the competitor, *b* = −0.131, 95% CI = [−0.228, −0.034], *p* = .008.

The ASC group were therefore less influenced than control participants by the decoy even when they had never seen the competing options before. This pattern indicates a reduced influence of local context rather than an effect driven by memory or need for consistency. Notably, applying the same analysis to the data from our AQ study revealed no effect of AQ group on the tendency to choose the decoy, *b* = −0.056, 95% CI = [−0.328, 0.217], *p* = .689, or on the tendency to choose the target rather than the competitor, *b* = 0.001, 95% CI = [−0.103, 0.105], *p* = .980. Thus, consistent with our primary analysis, this analysis indicates that the difference in context sensitivity between people with ASC and control participants was more pronounced than the difference in context sensitivity between members of the general population with low and high AQ scores: The only effect of AQ group was on the tendency to make within-subject preference reversals, whereas, in comparison with the control group, the ASC group both made fewer preference reversals and showed a reduced tendency to select the target when a given stimulus pair was encountered for the first time.

### Noisy responding

Next, we tested whether the enhanced consistency of the ASC group and the high-AQ group was driven by a reduction in random responding rather than an altered sensitivity to contextual stimuli (Pettibone, 2012). This analysis and the next were not part of our preregistered analysis strategy but seemed like useful explorations.

We computed the proportion of decoy selections across the 20 test trials for each participant as an index of noisy responding (Pettibone, 2012) and reran the regression analysis that contrasted consistent with inconsistent choices with this noisy-responding measure included as a predictor. The results were virtually identical to those of the original analyses: The ASC individuals in the ASC study made more consistent choices (fewer preference reversals) than did the control participants, *b* = 0.188, 95% CI = [0.047, 0.329], *p* = .010, and the high-AQ group in the AQ study made more consistent choices than did the low-AQ group, *b* = 0.157, 95% CI = [0.010, 0.304], *p* = .037. The index of noisy responding only weakly predicted the tendency to make consistent choices rather than preference reversals (ASC study: *b* = −0.130, 95% CI = [−0.277, 0.017], *p* = .082; AQ study: *b* = −0.124, 95% CI = [−0.263, 0.014], *p* = .078). The reduced attraction effect among the ASC group and the high-AQ group is therefore unlikely to have been due to a change in random or inattentive choice.

### Response times

Finally, we examined whether the greater consistency of the ASC group was due to slower, more deliberative de-cision making. We computed each participant’s mean re-sponse time (RT) across the 20 test trials and log-transformed these means to normalize the data and reduce the influence of extreme values. We examined the relationship between this measure and participant group with linear regression (including the same additional predictors as in our main analyses) and then assessed the association between RTs and choice behavior by rerunning our primary analysis with the RT variable added as a predictor.

Participants with ASC had longer response latencies than control participants (ASC group: geometric mean = 24.3 s, 95% CI = [21.9, 27.1]; control group: geometric mean = 17.8 s, 95% CI = [16.3, 19.5]; *b* = 0.088, 95% CI = [0.014, 0.163], *p* = .020). However, the effects of ASC on choice behavior were not altered by including RT as a predictor: ASC participants remained more likely than control participants to make consistent choices, *b* = 0.189, 95% CI = [0.048, 0.331], *p* = .009, and were less likely than control participants to make decoy selections, *b* = −0.249, 95% CI = [−0.495, −0.001], *p* = .049. In addition, although participants with shorter RTs were more likely to make decoy selections and non-attraction preference reversals (decoy selections: *b* = −0.604, 95% CI = [−0.811, −0.396], *p* < .001; non-attraction preference reversals: *b* = 0.513, 95% CI = [0.172, 0.853], *p* = .003), there was no meaningful association between response latency and the tendency to make consistent choices rather than preference reversals, *b* = 0.079, 95% CI = [−0.059, 0.216], *p* = .263.

Similar results emerged in the AQ study. The response latencies of the low-AQ and high-AQ groups were very similar to one another (low-AQ group: geometric mean = 17.7 s, 95% CI = [16.2, 19.3]; high-AQ group: geometric mean = 16.8 s, 95% CI = [15.7, 18.0]; *b* = −0.031, 95% CI = [−0.085, 0.024], *p* = .269), and although participants with shorter RTs were more likely to make decoy selections, *b* = −0.303, 95% CI = [−0.521, −0.083], *p* = .007, there was no association between response latency and the tendency to make consistent choices, *b* = −0.001, 95% CI = [−0.137, 0.135], *p* = .990, and controlling for response latency made very little difference to the effect of AQ group on choice consistency, *b* = 0.161, 95% CI = [0.013, 0.309], *p* = .033.

In short, participants who rushed their decisions were more likely to make random responses, but there is no indication that the reduced context sensitivity of people with high AQ scores or with ASC was a consequence of their taking longer over their choices. Their increased decision time is consistent with research showing that people with ASC are reluctant to make decisions at all, and do not simply take a more deliberative strategy than neurotypical individuals (Luke et al., 2012).

## Discussion

People with autism spectrum conditions made fewer context-induced preference reversals than did neurotypical individuals. That is, they made more conventionally rational decisions. Our results accord with evidence of reduced loss/gain framing effects when people with ASC make choices between gambles (De Martino, Harrison, Knafo, Bird, & Dolan, 2008) and extend the extensive demonstrations of reduced sensitivity to global context in perceptual and cognitive tasks to a new domain: ASC participants were more likely than control participants to represent the value of each attribute or option in isolation, rather than being influenced by the other items in the choice set. This kind of reduced context sensitivity has traditionally been labeled *weak central coherence*—a diminished ability to integrate local information into a global gestalt (Frith, 1989). However, the original conception of weak central coherence does not capture enhanced choice consistency in a “high-level” decision task such as ours, in which there is no global percept. Rather, our data support more recent suggestions that autism is characterized by a wide-ranging enhancement of, or preference for, local information processing (e.g., Happé & Frith, 2006; Plaisted et al., 2003).

Why were people with ASC less susceptible than control participants to context effects in our choice task? There are many accounts proposing mechanisms for context-induced preference reversals (see Howes, Warren, Farmer, El-Deredy, & Lewis, 2016, for a recent review). Two are of particular relevance to ASC. The first posits that choices are based on how readily they can be justified, “even when there is no overt need to justify to others” (Simonson, 1989, p. 159; see also Pettibone & Wedell, 2000). The target is better than the decoy on both dimensions (whereas the competitor is superior on only one), and this provides a reason to choose the target option, increasing its choice share (Simonson, 1989). Consistent with this, the target is rated as more justifiable than the competitor (Pettibone & Wedell, 2000), and the attraction effect increases when people believe that they will have to justify their decisions to other people (Simonson, 1989). The reduced context effect in people with ASC might therefore be a further manifestation of their reduced understanding of, or concern for, the likely beliefs and appraisals of others (Baron-Cohen, 2002). However, this framework lacks formalization, does not capture a full spectrum of context effects, and cannot readily account for data from nonhuman species (e.g., Lea & Ryan, 2015).

A more precise and general mechanism for a wide range of context effects is offered by the *multiattribute linear ballistic accumulator* model (Trueblood et al., 2014), which posits a linear rise to threshold of units whose drift rates reflect the value of the corresponding option relative to the other items in the set. More difficult discriminations attract more attention, so the similarity between the target and the decoy means that the target benefits from its favorable comparison with the decoy more than the competitor does; this produces the attraction effect. Correspondingly, an increase in the discriminability of the target and distractor will reduce the preferential attention to the decoy-target comparison and weaken the effect. People with ASC have been found to show enhanced discrimination on a range of perceptual tasks, and this observation has led to the proposal that autism is characterized by an enhanced sensitivity to differences and a reduced processing of common features (Plaisted et al., 2003). There is debate about the generality of enhanced discrimination across tasks and domains (Happé & Frith, 2006) and about the neural mechanisms involved (Davis & Plaisted-Grant, 2015), but a greater separation of attribute values in representational space among people with ASC, compared with neurotypical individuals, provides an explanation for our findings that links a wide-ranging model of context effects to a diverse body of empirical and theoretical work on autism.

Recent work with neurotypical adults has also considered the functional basis for the attraction effect. Whereas conventional accounts of rational choice dictate choice consistency, emerging Bayesian frameworks construe preference reversals as an adaptive response to uncertainty about the value of an option. One account casts the attraction effect as a rational inference about the trade-off between attribute dimensions in the marketplace (Shenoy & Yu, 2013). A more general model proposes that a decision maker’s estimate of the utilities of alternatives can be improved by using prior estimates based on the ordinal relations between the attributes (Howes et al., 2016). The mathematical specification of this model is complex, but the core principle is that decision making is improved when noisy computations of expected value are supplemented by considering the ordering of attribute values: Options whose attributes have higher-rank positions on the relevant dimensions usually have higher expected value, and the attraction-effect decoy increases the rank position of the target (but not the competitor) on its worst dimension, so it is rational to infer that the target is the better option.

These ideas link to developments in theorizing about autism. In particular, Pellicano and Burr (2012) have recently proposed that autism is characterized by unusually flat priors (*hypopriors*), such that perception is driven by current sensory input that is little influenced by background expectations. Within the Bayesian framework for the effects of context on choice, such hypopriors would entail reduced reliance on the ordinal relations between option attributes and, correspondingly, a reduced tendency to make context-induced preference reversals—as we observed. Thus, autistic traits may allow people to avoid the potentially biasing effect of context by sacrificing useful information about the likely state of the world, given past experience.

Our results suggest several avenues for future research. Straightforward steps include examining whether the more conventionally rational responses of ASC participants extend to other types of context effect, such as the compromise effect (Simonson, 1989) and gambler’s fallacy (Kahneman & Tversky, 1972); exploring the effects of moderating variables, such as time pressure (Pettibone, 2012); and using process-tracing techniques such as eye tracking to uncover how autistic traits shape the processes by which options are sampled and compared (N. Stewart, Hermens, & Matthews, 2016). More broadly, most studies of decision making in participants with neuropsychiatric conditions, such as ASC, have focused on how people learn, represent, and evaluate probabilities and rewards (e.g., Damiano, Aloi, Treadway, Bodfish, & Dichter, 2012). The present results show that choice behavior may be atypical even when people simply select the best alternative from explicitly stated, risk-free outcomes. Studying the context sensitivity of such choices has the potential to reveal new insights into the decision-making strategies of individuals with a range of neuropsychological conditions.

Beyond these theoretical and empirical directions, the present findings have practical implications for the socioeconomic functioning of people with ASC. The attraction effect influences elections (Pan, O’Curry, & Pitts, 1995), legal judgments (Kelman, Rottenstreich, & Tversky, 1996), and policy decisions (Herne, 1997). The ability of decoys to shape consumer behavior has been observed in field studies (Doyle et al., 1999) and real-world marketing campaigns (Ariely, 2009). Our data suggest that people with autistic traits are still influenced by such decoys but that the effect is smaller for them than for the general population. This reduction in the attraction effect offers some protection against the biases that can result from context-induced preference. However, the price that people with autistic traits pay for this resistance to contextual influence may be a reduction in the potentially adaptive updating of beliefs about optimum choice that comes from using local comparisons to inform decision making.

## Supplementary Material

Supplementary material

Supplementary material
