# Supplementary material for: People With Autism Spectrum Conditions Make More Consistent Decisions
Source: Psychol Sci. 2017 Jun 21;28(8):1067–76. doi: 10.1177/0956797617694867 (PMC5548251; doi:10.1177/0956797617694867)

Supplementary Materials

Table S1. Product categories and attribute values used in the ASC study and in Version 1 of the AQ study. D_A is the decoy that renders option A the target; D_B is the decoy that targets option B.

**Product**	**Attributes**	**A**	**B**	**D_A**	**D_B**
Cell phone	Number of apps	16	32	12	28
	Repair rate (%)	3	5	3.5	5.5
USB drive	Capacity (GB)	16	32	12	28
	Lifespan (months)	36	20	32	16
Paper towels	Strength (0-10)	4	8	3	7
	Absorbency (millilitres)	52	28	46	22
Orange juice	Vitamin C (mg)	34	82	22	70
	Calories (Kcal)	33	69	42	78
Apartment	Size (Square feet)	759	1203	648	1092
	Crime rate (per month)	10	15	11	16
Printer	Cost (cents per page)	7.05	3.61	7.91	4.47
	Speed (pages per minute)	16.7	5.9	13.3	1.5
Headphones	Sound quality (0-100)	68	92	62	86
	Lifespan (months)	24	12	20	10
Highlighter pen	Brightness (0-1)	0.4	0.8	0.3	0.7
	Volume (millilitres)	180	100	160	80
Walking shoes	Durability (months)	8	32	2	26
	Comfort (0-100)	88	64	82	58
Part-time job	Wages ($ per hour)	6.60	8.20	6.20	7.80
	Commuting time (minutes)	20	60	30	70

Table S2. Product categories and attribute values used in Version 2 of the AQ study. A_d is the decoy that renders option A the target; B_d is the decoy that targets option B.

**Product**	**Attributes**	**A**	**B**	**D_A**	**D_B**
Car	Safety (0-5)	4	5	3.8	4.8
	Efficiency (mpg)	49.65	31.05	44.25	25.65
Six-pack beer	Price ($)	6.79	3.19	7.22	3.62
	Quality (0-5)	4.10	2.50	3.80	2.20
Cell phone battery	Price ($)	27.50	19.00	30.00	21.50
	Talk time (hours)	14	11	13	10
Restaurant	Atmosphere (0-100)	76	96	72	92
	Food (0-100)	95	74	90	69
Digital camera	Screen Size (inches)	1.2	3	1	2.8
	Zoom (magnification)	10.1x	4.1x	9x	3.0x
Light bulb	Lifetime (hours)	1000	2000	980	1980
	Price ($)	1.20	2.40	1.50	2.70
Mouthwash	Fresh Breath (hours)	6	12	5	11
	Volume (fluid ounces)	25	16	23	14
Television	Screen size (inches)	40	65	38	63
	Picture quality (0-100)	6.23	4.63	5.5	3.9
Internet provider	Average Speed (Kb per second)	386	646	290	550
	Download limit (GB)	292	150	270	128
Language course	Number of Lessons	10	18	8	16
	Price ($)	30	50	35	55

Table S3. Comparison of the Control and ASC samples

**Comparison**	**ASC**	**Control**	**Test Statistic**	***p***
N	90	212	-	-
Males / Females *	37 / 52	89 / 123	χ^2 = 0.004	.950
Residence: UK / US *	70 / 18	169 / 43	χ^2 = 0.001	.975
Mean Age (SD)	43.11 (13.73)	43.88 (13.55)	*t* = -0.445	.657
Mean ICAR (SD)	10.83 (3.98)	7.25 (3.66)	*t* = 7.328	< .001
Mean AQ (SD)	92.50 (10.57)	65.20 (10.04)	*t* = 20.840	< .001

*One person in the ASC group preferred not to state their gender. Two people in the ASC group did not indicate their country of residence. The chi-square tests exclude these participants.

Table S4. Correlations between demographic variables for the ASC group. Asterisks indicate a significant correlation, *p*<.05.

	**Age**	**ICAR**	**AQ**
Age			
ICAR	-.03		
AQ	.00	.01	
Gender	.39*	.04	.02

Table S5. Correlations between demographic variables for the Control group. Asterisks indicate a significant correlation, *p*<.05.

	**Age**	**ICAR**	**AQ**
Age			
ICAR	-.03		
AQ	.05	.22*	
Gender	.39*	.04	.11

Table S6. Means for ICAR, age, and AQ in the high and low AQ groups for the two versions of the AQ study.

	Males/ Females	Mean (SD)		
		**Age**	**AQ**	**ICAR**
**Version 1**				
Low AQ	44/37	35.42 (11.43)	48.59 (3.97)	7.98 (3.39)
High AQ	58/36	37.01 (11.79)	86.02 (5.53)	9.46 (3.33)
**Version 2**				
Low AQ	41/53	35.99 (12.27)	50.23 (3.43)	8.36 (3.57)
High AQ	54/46	34.17 (8.92)	84.51 (5.16)	9.59 (3.33)
**Combined**				
Low AQ	85/90	35.73 (11.86)	49.48 (3.77)	8.18 (3.48)
High AQ	112/82	35.55 (10.48)	85.24 (5.38)	9.53 (3.32)

Table S7. Comparison of the high and low AQ samples (collapsed across study version)

**Comparison**	**High**	**Low**	**Test Statistic**	***p***
n	194	176	-	-
Males / Females *	112/82	85 /90	χ^2 = 2.745	.100
Mean Age (SD)	35.55 (10.48)	35.73 (11.86)	*t* = -0.155	.877
Mean ICAR (SD)	9.53 (3.32)	8.18 (3.48)	*t* = 3.790	< .001
Mean AQ (SD)	85.24 (5.38)	49.48 (3.77)	*t* = 74.572	< .001

*One person in the low AQ group preferred not to state their gender. The chi-square test excludes this participant.

Table S8. Correlations between demographic variables for the low-AQ group. Asterisks indicate a significant correlation, $p<.05$.

	**Age**	**ICAR**	**AQ**
Age			
ICAR	.06		
AQ	.06	-.08	
Gender	-.16*	.04	.02

Table S9. Correlations between demographic variables for the high-AQ group. Asterisks indicate a significant correlation, $p<.05$.

	**Age**	**ICAR**	**AQ**
Age			
ICAR	.08		
AQ	-.02	.15*	
Gender	-.22*	.07	-.08

Table S10. Response proportions by gender for the ASC participants. The columns show the mean proportion of choices of each type and the results of a between-subject Welch-corrected t-test that compares the two genders. The 95% CIs for all estimated effect sizes span zero.

	M_{males} (SD)	$M_{females}$ (SD)	*t*(df)	*p*	d [95% CI]
Decoy-selection	.022 (.048)	.037 (.079)	1.10 (85.06)	.273	-0.22 [-0.65, 0.21]
Consistent	.757 (.176)	.706 (.202)	1.27 (83.52)	.209	0.27 [-0.16, 0.70]
Attraction	.192 (.161)	.242 (.182)	1.38 (92.95)	.171	-0.29 [-0.72, 0.14]
Non-attraction	.030 (.062)	.015 (.041)	1.23 (58.55)	.224	0.28 [-0.15, 0.71]

Figure S1. ASC study: Primary regression analysis

The panels show the regression coefficients for the mixed-effects logistic regression analyses described in the main text. Here and for all other plots of regression coefficients, the error bars show 95% Wald confidence intervals and black data points indicate significant effects (*p* <.05, two-tailed). The panels show the coefficients for three contrasts: decoy-selection vs any other outcome, consistent choice vs preference reversal, and attraction-effect preference reversal vs non-attraction preference reversal. (BIC values: 1531.7, 3390.0, 637.9, respectively.)

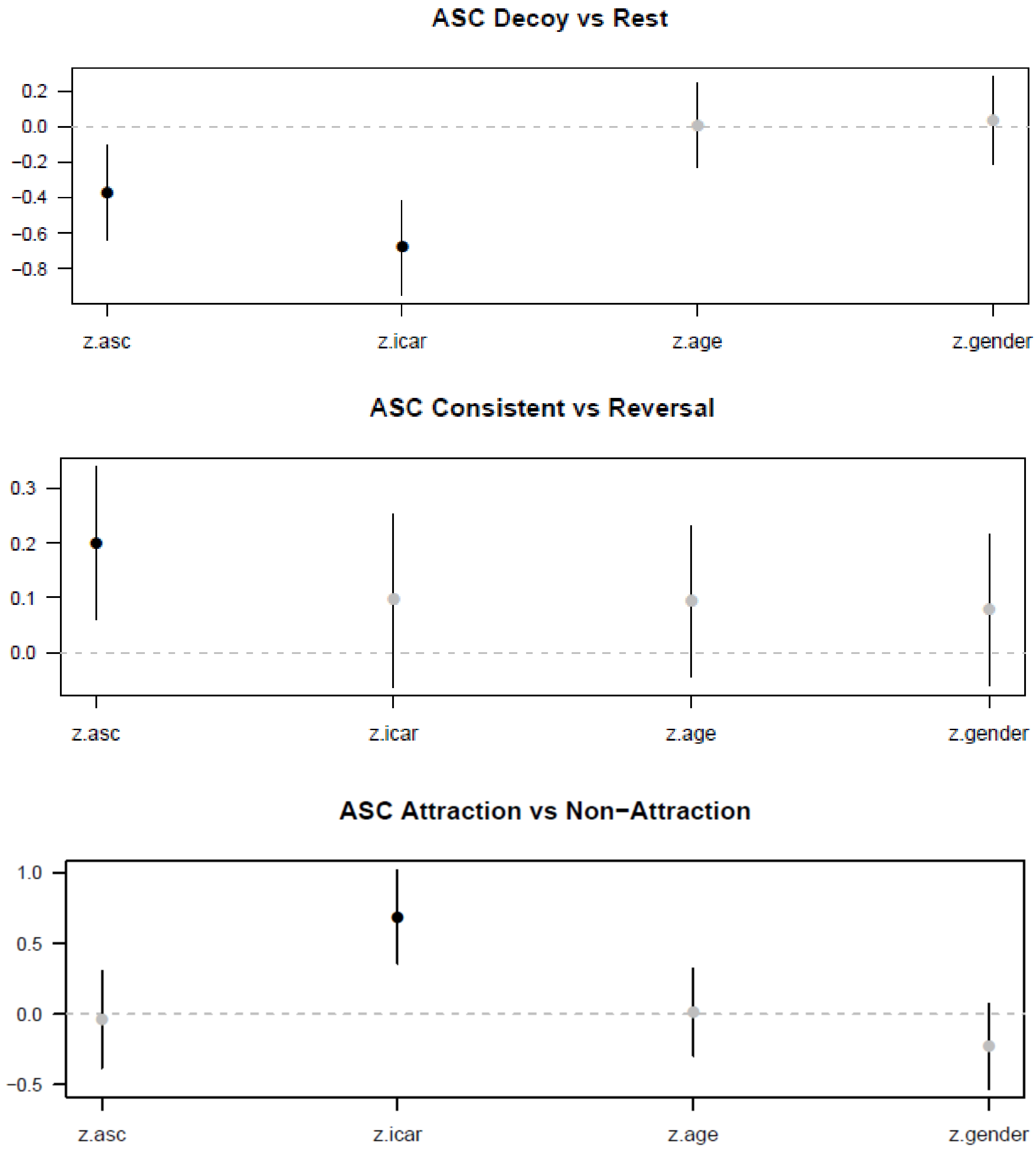

Figure S2. ASC study: First choice proportions

The plot shows the proportions of times participants in the ASC and neurotypical (NT) control group chose the target, competitor, and decoy options on the first presentation of each product pair.

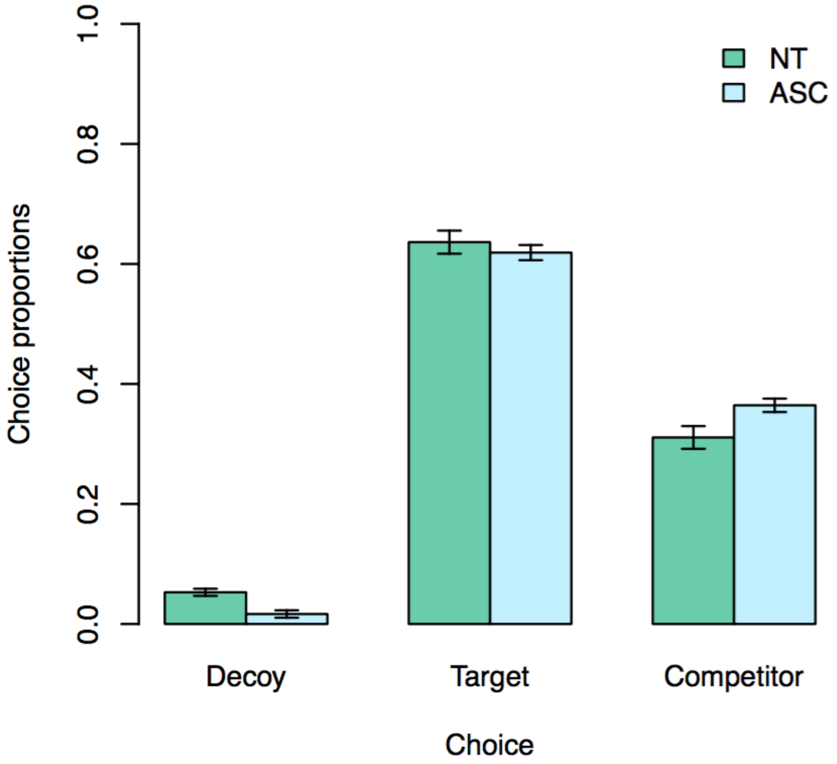

Figure S3. ASC study: Regression analysis of first choices

The panels show the results of analysing participants' responses on the first occurrence of each product pair. The top panel shows the coefficients from contrasting the tendency to choose the decoy (coded 1) with the tendency to choose one of the other options (target or competitor, both coded 1); the bottom panel plots the coefficients obtained when contrasting target choices (coded 1) against competitor choices (coded 0). (BIC values 1059.2 and 3774.6, respectively).

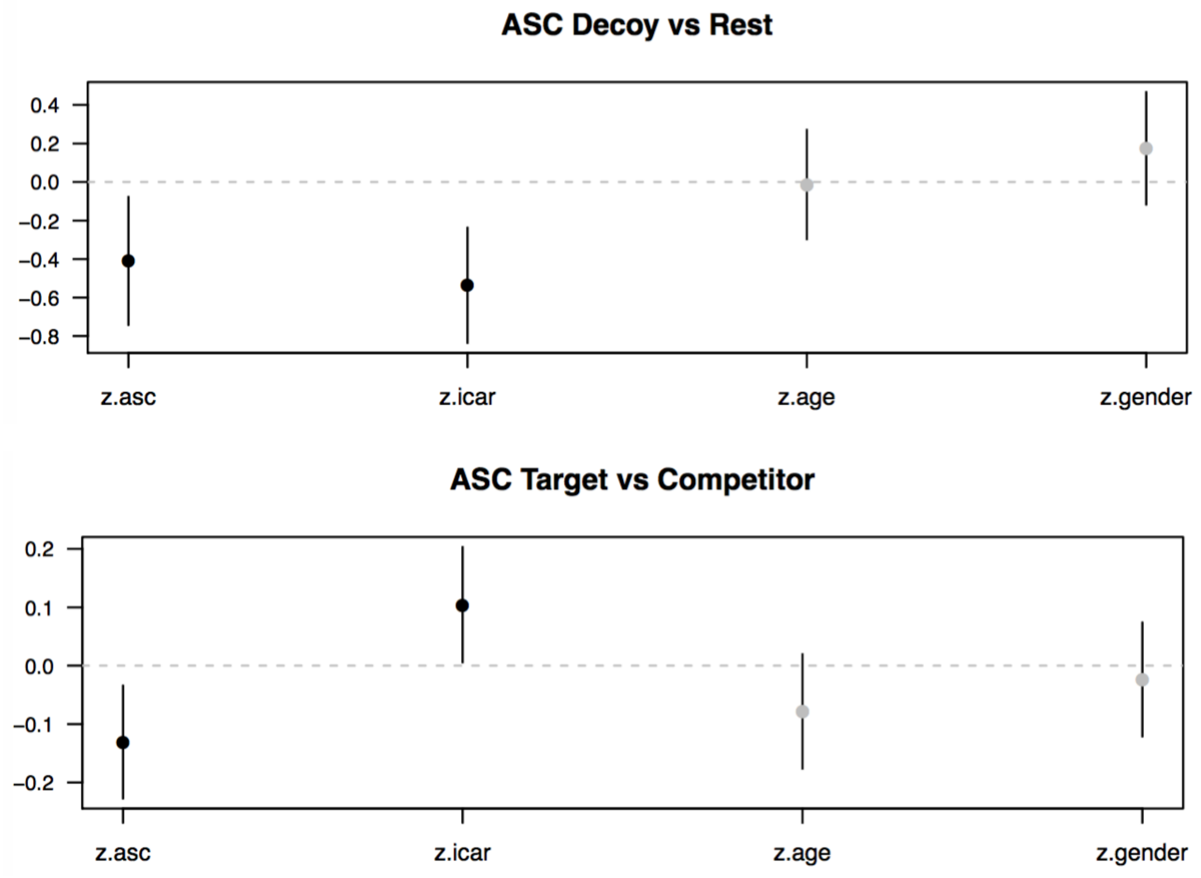

Figure S4. ASC study: Controlling for random responding

The panels show the results of re-running the primary analysis controlling for random responding, indexed by the participant's proportion of decoy selections across the 20 test trials (pdecoy). Note that it would not make sense to include pdecoy in the Decoy vs Non-decoy contrast, so only the Consistent Choice vs Preference Reversal and Attraction-Effect Preference Reversal vs Non-attraction Preference Reversal contrasts are analysed; BIC values: 3402.9 and 644.4, respectively.

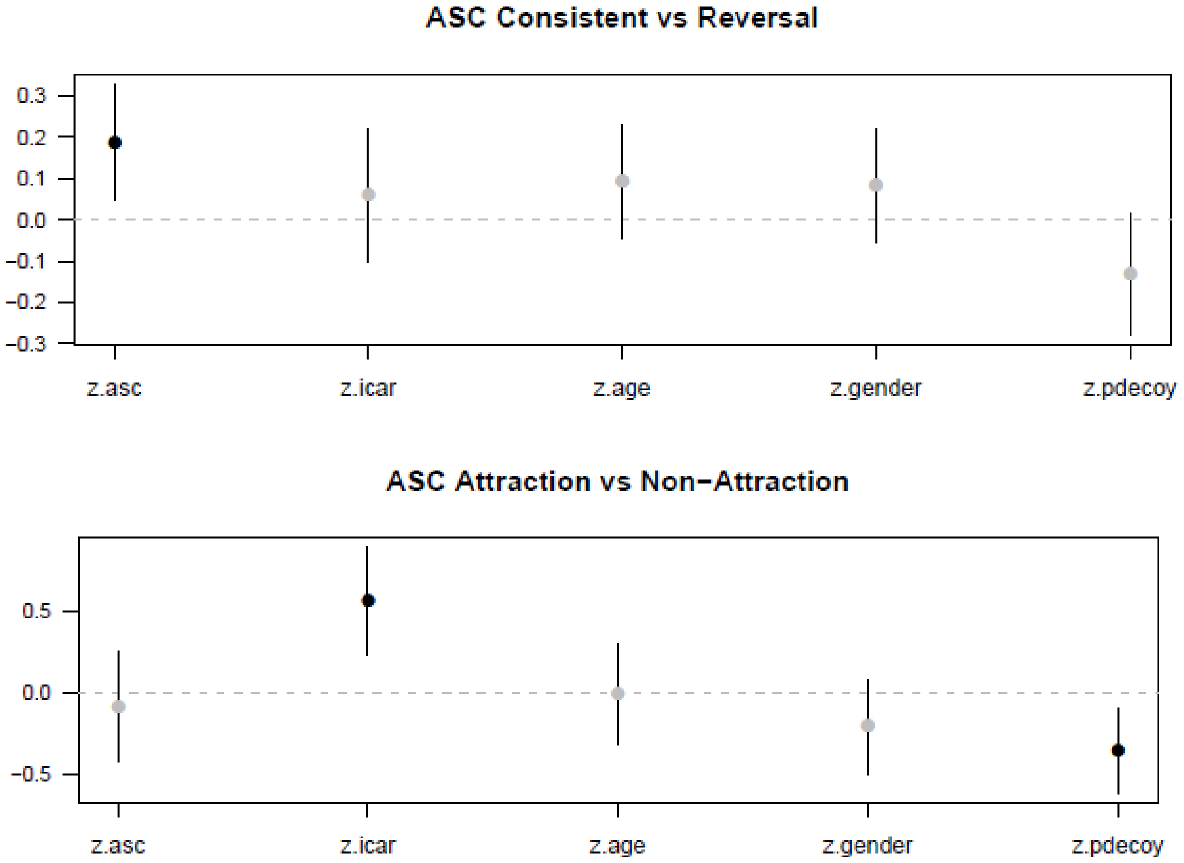

Figure S5. ASC study: Comparing decision times

As described in the main text, for our analysis of decision-times we tested whether the ASC and Control groups differed in the mean response time by running a linear regression. The plot shows the regression coefficients for this analysis (adjusted R-sq =.089).

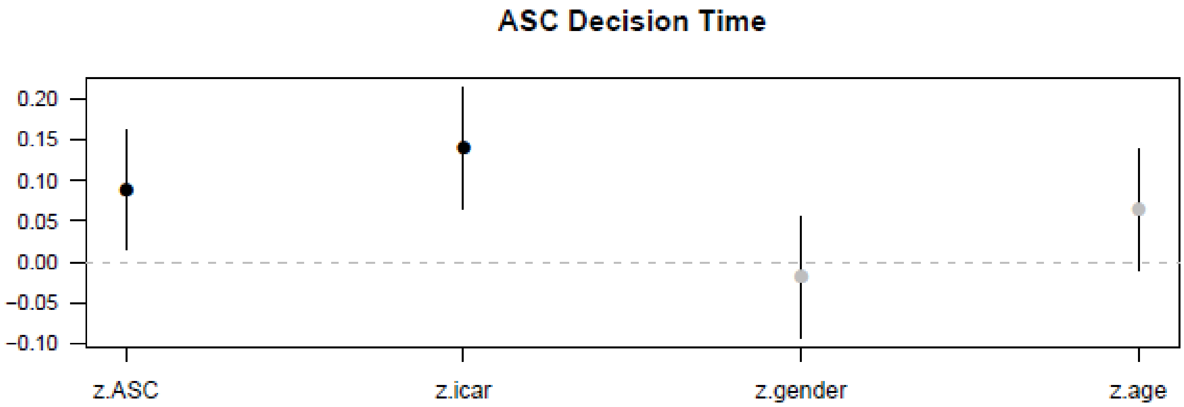

Figure S6. ASC study: Controlling for decision times

The panels show the results of re-running the primary analysis with each person's log-transformed mean response-time (logmt) as an additional predictor. (BIC values 1516.3, 3404.7, and 641.1 for top, middle, and bottom analyses, respectively.)

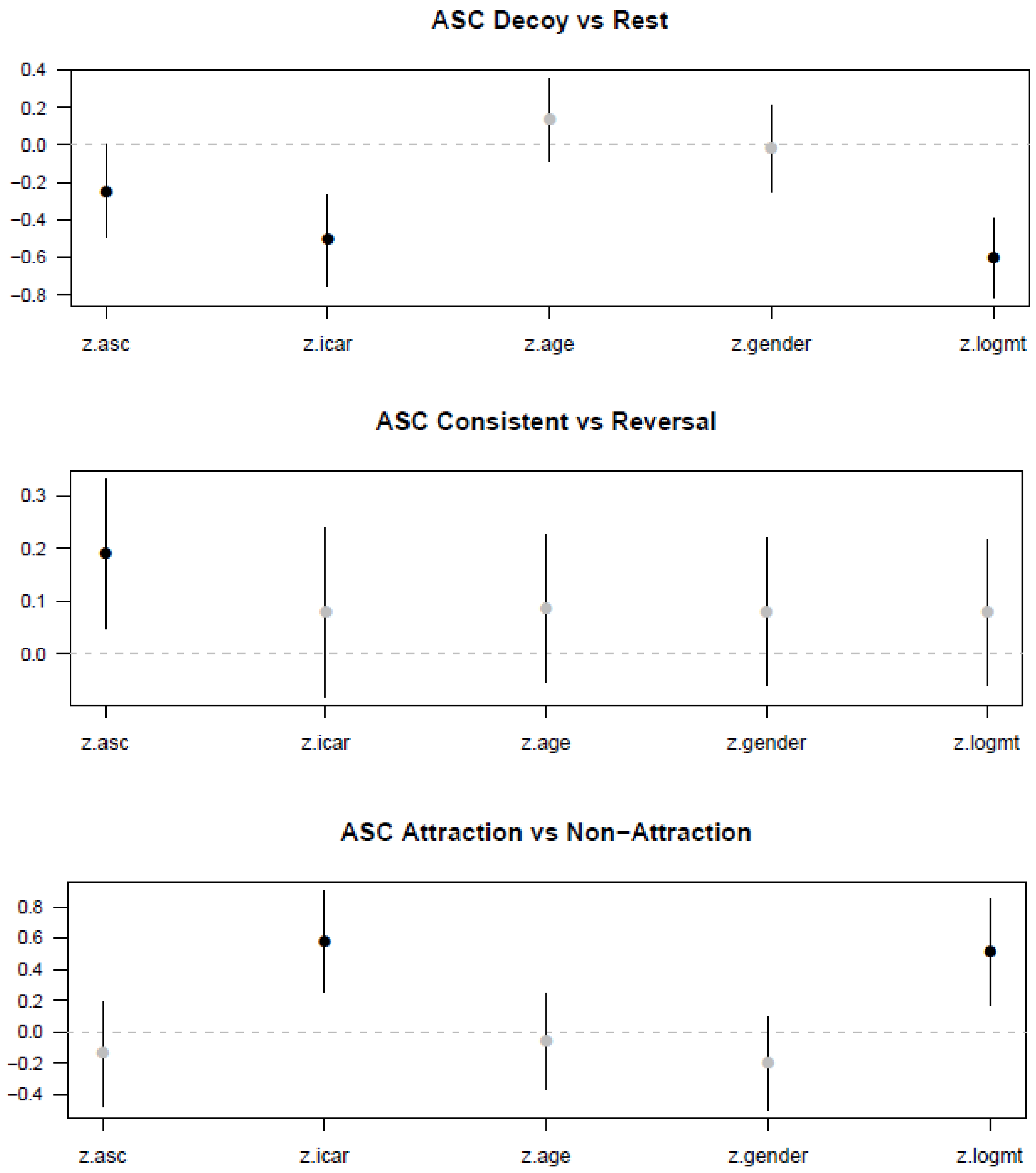

Figure S7. AQ study: Primary regression analysis

The panels show the regression coefficients for the three contrasts described in the main text. As reported in the Methods section, the analyses included the Version variable (Version 1 coded 0; Version 2 coded 1) and the interaction between Version and all other variables to examine the consistency of the findings across participant samples / stimulus sets. As for other variables, Version was standardized prior to each regression, and the interaction terms were computed by multiplying the standardized predictors (e.g., the coefficient labelled aq.int is z.aq*z.version). (BIC values for the top, middle, and bottom analyses are: 1655.2, 3959.4, and 703.7, respectively).

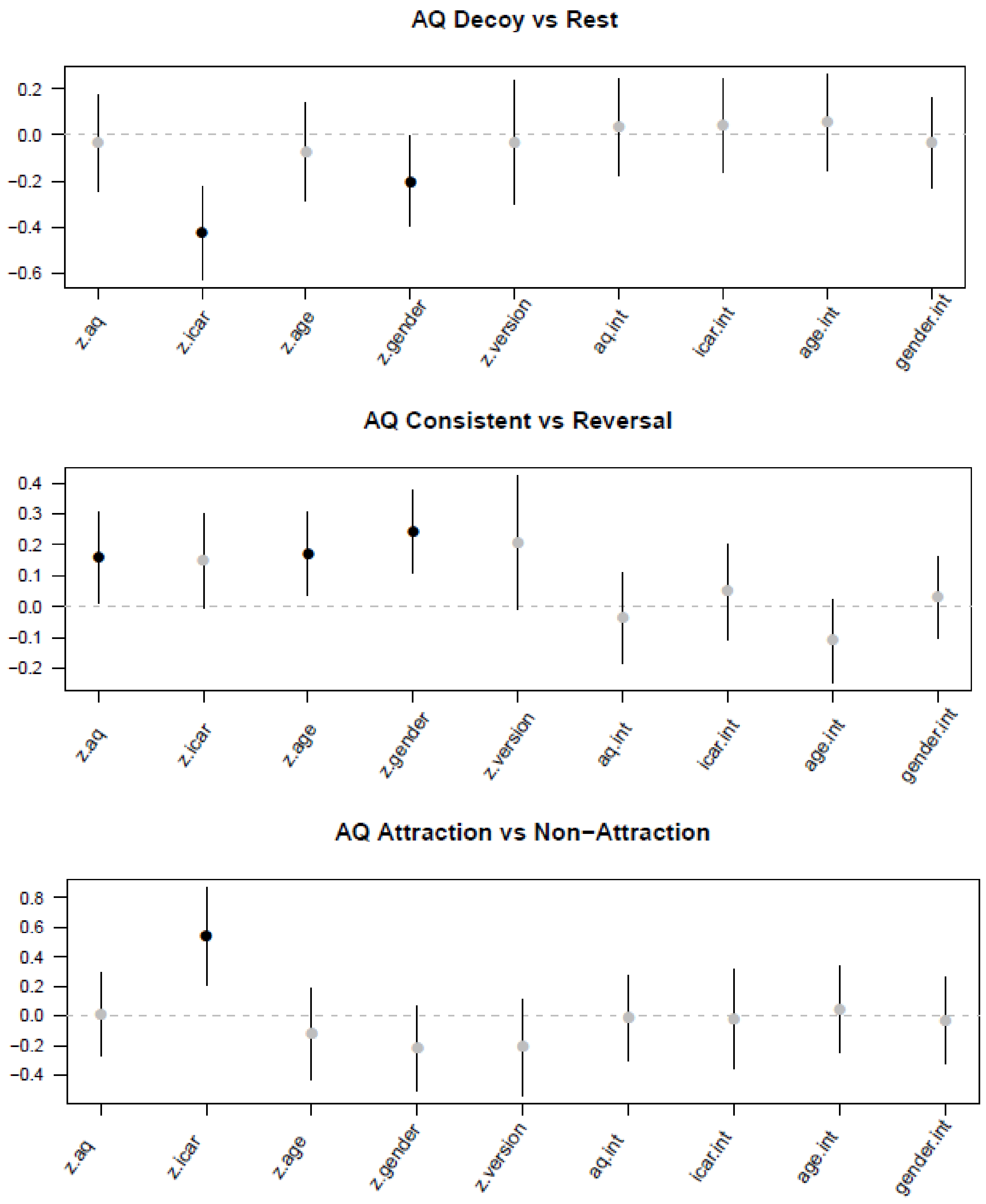

Figure S8. AQ study Version 1: Primary regression analysis

The panels show the regression coefficients for the three contrasts described in the main text, limited to the data from the first version of the study. (BIC values for the top, middle, and bottom analyses are: 815.8, 1974.5, and 349.8, respectively).

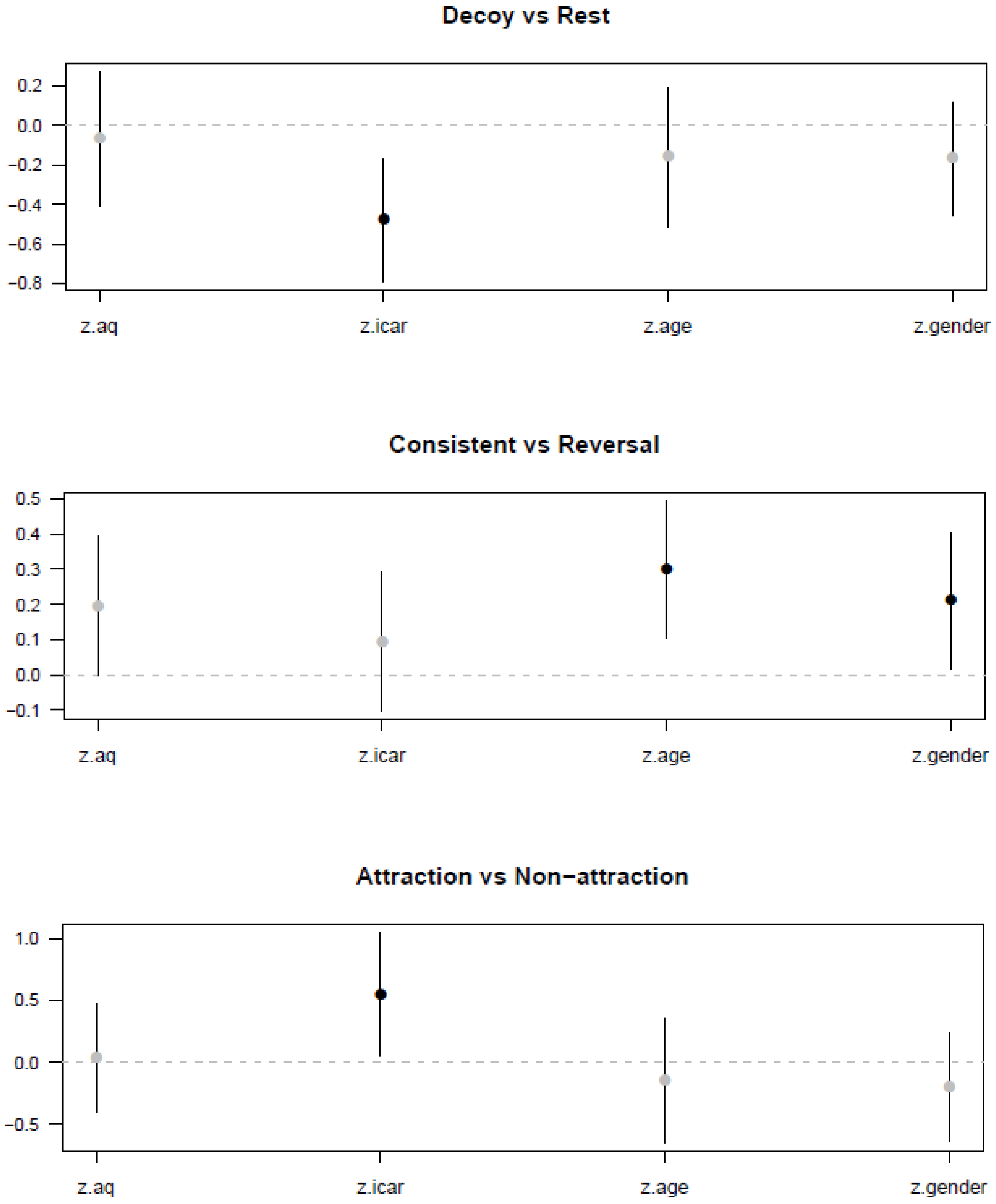

Figure S9. AQ study Version 2: Primary regression analysis

The panels show the regression coefficients for the three contrasts described in the main text, limited to the data from the second version of the study. (BIC values for the top, middle, and bottom analyses are: 871.2, 2012.3, 379.4, respectively).

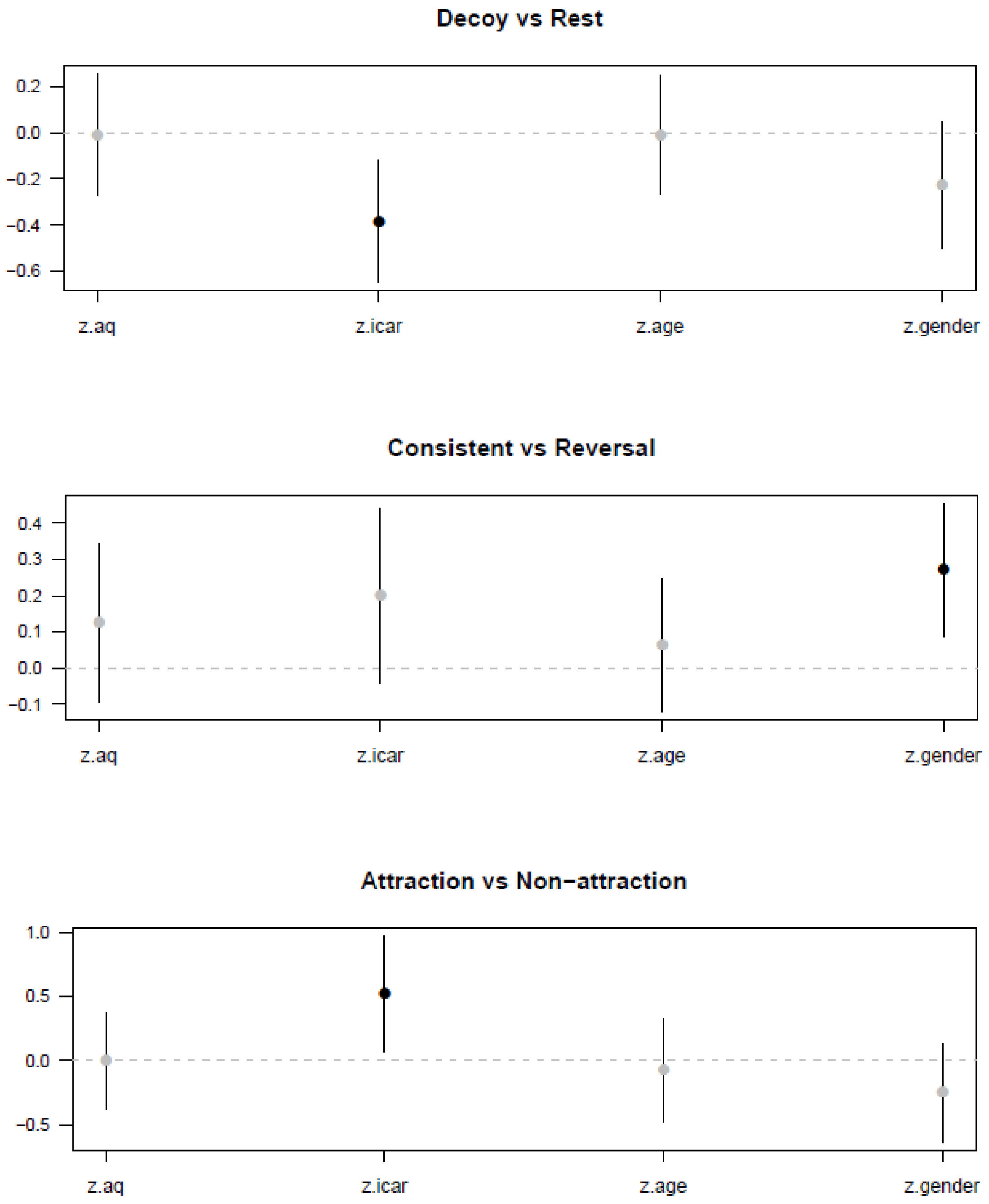

Figure S10. AQ study: First choice proportions

The plot shows the proportions of times participants in the low- and high-AQ groups chose the target, competitor, and decoy options on the first presentation of each product pair.

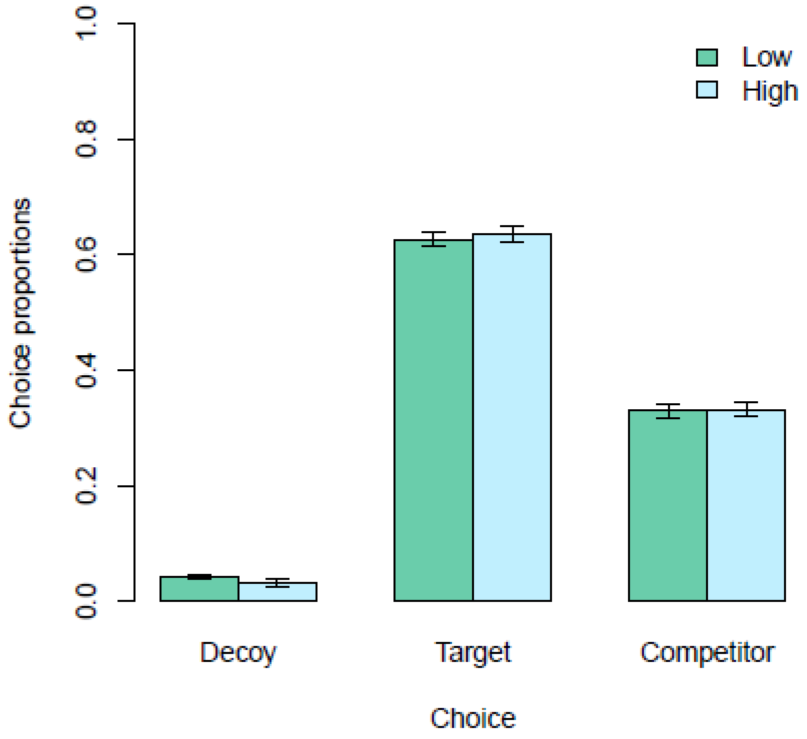

Figure S11. AQ study: Regression analysis of first choices

The panels show the results of analysing participants' responses on the first occurrence of each product pair. The top panel shows the coefficients from contrasting the tendency to choose the decoy (coded 1) with the tendency to choose one of the other options (target or competitor, both coded 1); the bottom panel plots the coefficients obtained when contrasting target choices (coded 1) against competitor choices (coded 0). (BIC values 1257.2 and 4651.2, respectively).

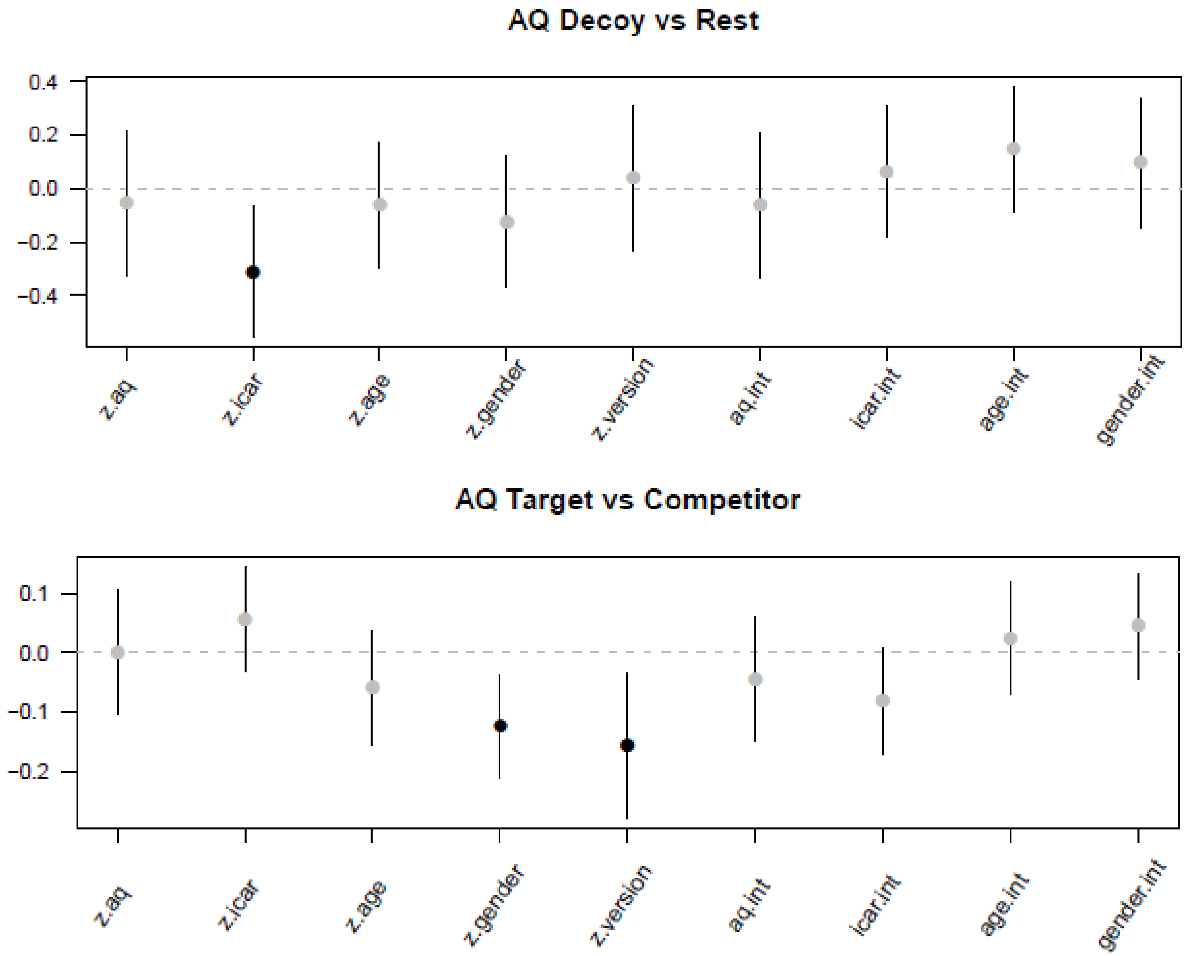

Figure S12. AQ study: Controlling for random responding

The panels show the results of re-running the primary analysis controlling for random responding, indexed by the participant's proportion of decoy selections across the 20 test trials (pdecoy). Note that it would not make sense to include pdecoy in the Decoy vs Non-decoy contrast, so only the Consistent Choice vs Preference Reversal and Attraction-Effect Preference Reversal vs Non-attraction Preference Reversal contrasts are analysed; BIC values: 3980.2 and 703.7, respectively.

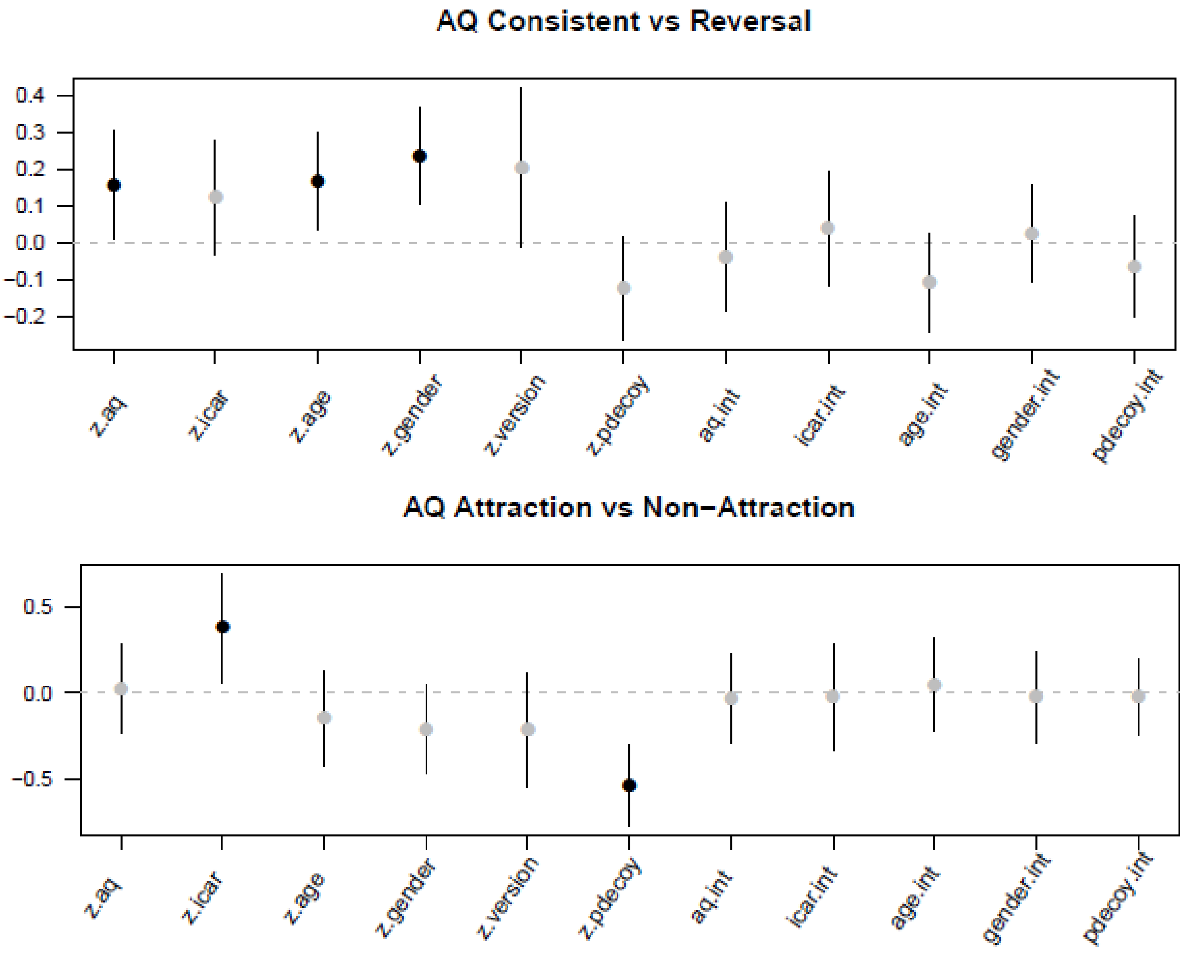

Figure S13. AQ study: Comparing decision times

As described in the main text, for our analysis of decision-times we tested whether the low-AQ and high-AQ groups differed in the mean response time by running a linear regression. The plot shows the regression coefficients for this analysis (adjusted R-sq = .088).

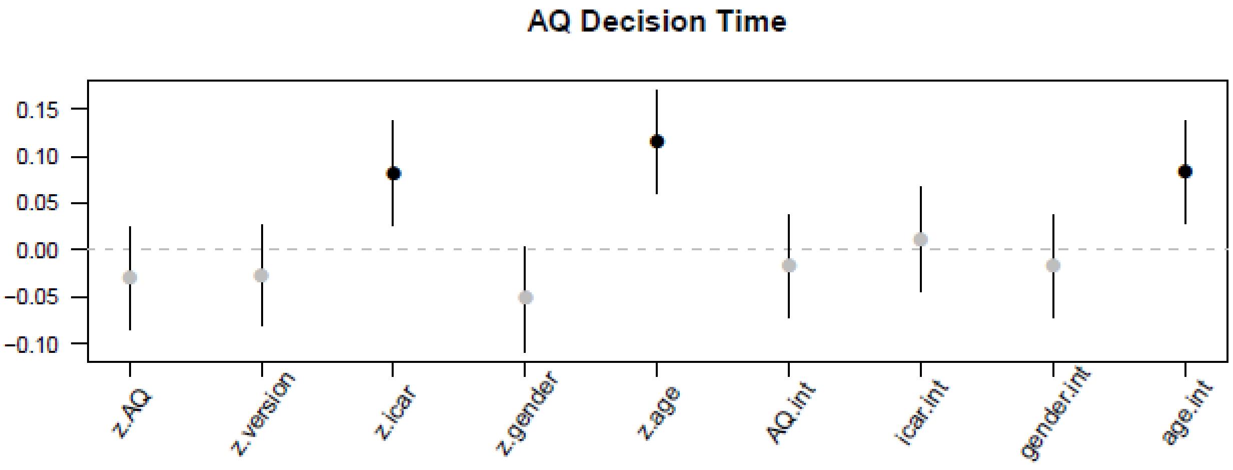

Figure S14. AQ study: Controlling for decision times

The panels show the results of re-running the primary analysis with each person's log-transformed mean response-time (logmt) as an additional predictor. (BIC values 1670.8, 3983.2, and 717.2 for top, middle, and bottom analyses, respectively.)

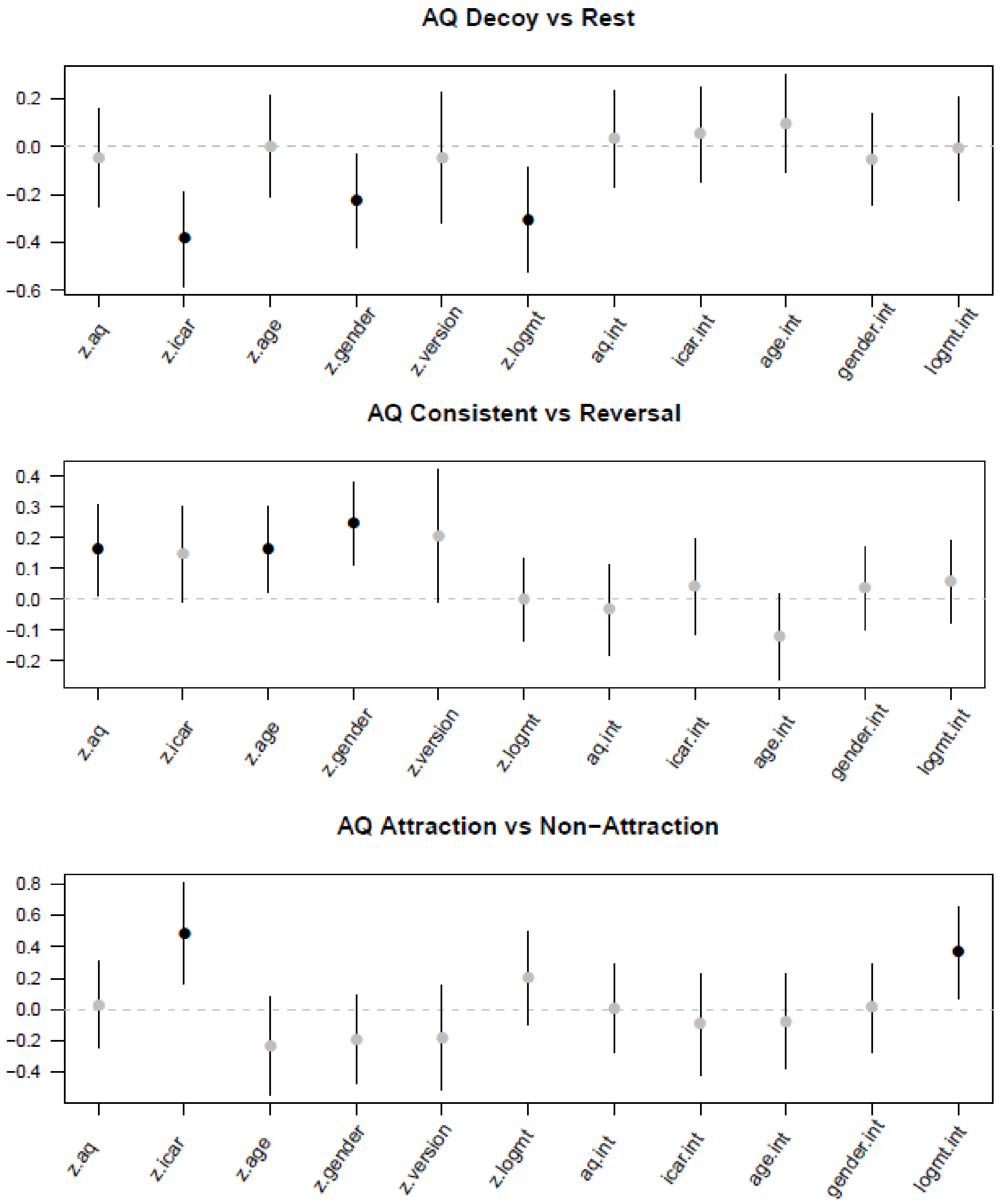

Supplement: Supplementary material [file Skylark_Supplemental_Material.pdf]
